# Conversion of methane to organic acids is a widely found trait among gammaproteobacterial methanotrophs of freshwater lake and pond ecosystems

**DOI:** 10.1128/spectrum.01742-23

**Published:** 2023-10-20

**Authors:** Ramita Khanongnuch, Rahul Mangayil, Antti Juhani Rissanen

**Affiliations:** 1 Faculty of Engineering and Natural Sciences, Tampere University, Tampere, Finland; 2 Department of Bioproducts and Biosystems, School of Chemical Engineering, Aalto University, Espoo, Finland; 3 Natural Resources Institute Finland, Helsinki, Finland; University of Minnesota Twin Cities, St. Paul, Minnesota, USA

**Keywords:** greenhouse gas, climate change, food web, extracellular metabolites, bioconversion, psychrophilic, psychrotolerant

## Abstract

Aerobic gammaproteobacterial methanotrophs (gMOB) are key organisms controlling methane fluxes at the oxic-anoxic interfaces of freshwater ecosystems. Under hypoxic environments, gMOB may shift their aerobic metabolism to fermentation, resulting in the production of extracellular organic acids. We recently isolated a gMOB strain representing the *Methylobacter* spp. of boreal lake water columns (i.e., *Methylobacter* sp. S3L5C) and demonstrated that it converts methane to organic acids (acetate, formate, malate, and propionate) under hypoxic conditions. Annotation for putative genes encoding organic acid production within the isolate’s genome and in environmental metagenome-assembled genomes (MAGs) representing *Methylobacter* spp. suggests that the potential for methane conversion into organic acids is widely found among *Methylobacter* spp. of freshwater ecosystems. However, it is not known yet whether the capability to convert methane to organic acids is restricted to *Methylobacter* spp. or ubiquitously present among other freshwater gMOB genera. Therefore, we isolated representatives of two additional gMOB genera from the boreal lake water columns, i.e., *Methylomonas paludis* S2AM and *Methylovulum psychrotolerans* S1L, and demonstrated similar bioconversion capacities. These genera could convert methane to organic acids, including acetate, formate, succinate, and malate. Additionally, S2AM produced lactate. Furthermore, we detected genes encoding organic acid production within their genomes and in MAGs representing *Methylomonas* spp. and *Methylovulum* spp. of lake and pond ecosystems. Altogether, our results demonstrate that methane conversion to various organic acids is a widely found trait among lake and pond gMOB, highlighting their role as pivotal mediators of methane carbon into microbial food webs of freshwater lake and pond ecosystems.

**IMPORTANCE:**

Aerobic gammaproteobacterial methanotrophic bacteria (gMOB) play an important role in reducing methane emissions from freshwater ecosystems. In hypoxic conditions prevalent near oxic-anoxic interfaces, gMOB potentially shift their metabolism to fermentation, resulting in the conversion of methane to extracellular organic acids, which would serve as substrates for non-methanotrophic microbes. We intended to assess the prevalence of fermentation traits among freshwater gMOB. Therefore, we isolated two strains representing relevant freshwater gMOB genera, i.e., *Methylovulum* and *Methylomonas*, from boreal lakes, experimentally showed that they convert methane to organic acids and demonstrated via metagenomics that the fermentation potential is widely dispersed among lake and pond representatives of these genera. Combined with our recent study showing coherent results from another relevant freshwater gMOB genus, i.e., *Methylobacter*, we conclude that the conversion of methane to organic acids is a widely found trait among freshwater gMOB, highlighting their role as pivotal mediators of methane carbon into microbial food webs.

## OBSERVATION

Gammaproteobacterial methanotrophs (gMOB) of several genera, e.g., *Methylobacter*, *Crenothrix*, *Methylomonas*, and *Methylovulum*, are key organisms controlling methane (CH_4_) fluxes at the oxic-anoxic interfaces of freshwater lake and pond ecosystems, where they can constitute over 50% of prokaryotes (1 – 3). Being obligate aerobic in nature, gMOB generally require O_2_ as the electron acceptor to oxidize CH_4_ into biomass and CO_2_. At the oxic-anoxic interface, however, they face fluctuating oxygen conditions and occasional hypoxia (i.e., oxygen limitation). During hypoxic conditions, gMOB may shift their cellular metabolism toward fermentation by generating various extracellular organic acids, as shown with a haloalkalitolerant strain *Methylotuvimicrobium alcaliphilum* 20Z (4). The CH_4_-derived organic acids could then serve as growth substrates for heterotrophic and methylotrophic bacteria in microbial food webs (5 – 7). Besides methanotroph biomass carbon, an important component of food webs until the top consumer level (8, 9), other microbes consuming CH_4_-derived soluble compounds potentially play a role in channeling CH_4_-carbon to consumers. We have recently shown that the potential for organic acid production is also found among freshwater lake gMOB (10). We isolated a psychrophilic gMOB strain representing genus *Methylobacter* (i.e., *Methylobacter* sp. S3L5C) from the water column of a boreal lake, demonstrated the bacterium’s capacity for the bioconversion of CH_4_ to organic acids, and predicted the putative genes (enzymes) driving this process (10). Furthermore, based on the analyses of metagenome-assembled genomes (MAGs), we concluded that the genetic potential to produce organic acids is a widely found trait among *Methylobacter* spp. in freshwater ecosystems (10 – 12), indicating their role as critical mediators regulating the bioconversion of CH_4_ to organic acids in freshwater ecosystems (5 – 7). However, to date, similar observations for other freshwater lake gMOB genera have not yet been reported. We hereby aim to demonstrate that the capability to convert CH_4_ to organic acids is not restricted to *Methylobacter* spp. but exists among other gMOB genera in freshwater lake and pond ecosystems.

**Fig 1:**
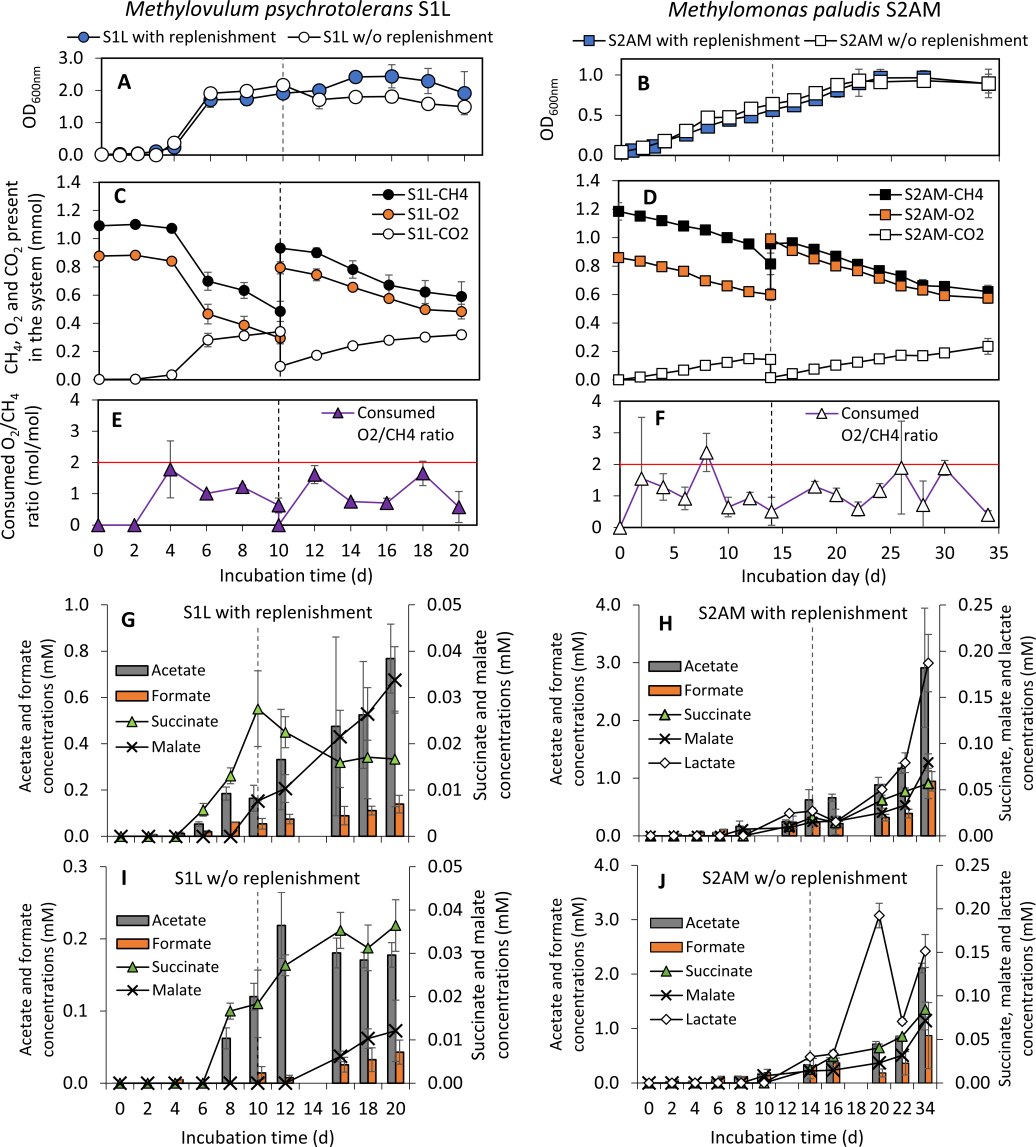
Performance on CH_4_ oxidation and organic acid excretion of *Methylovulum psychrotolerans* S1L (left) and *Methylomonas paludis* S2AM (right) during the test with and without CH_4_ and air replenishment on days 10 and 14 (vertical dot line) for strains S1L and S2AM, respectively. The profiles of (**A, B**) cell growth during the test with and without the gas replenishment, as well as the profiles of (**C, D**) CH_4_ and O_2_ utilization and CO_2_ production, and (**E, F**) consumed O_2_/CH_4_ molar ratio during the test with the gas replenishment. The red horizontal line (**E, F**) indicates the stoichiometric O_2_/CH_4_ ratio for aerobic methane oxidation (CH_4_ + 2O_2_ → CO_2_ + 2H_2_O). (**G, H**) Organic acid excretion profile during the test with the replenishment and (**I, J**) without the replenishment. The error bars represent the standard deviation among the biological triplicate samples.

To address our aim, we isolated representatives of two additional freshwater lake gMOB genera, i.e., *Methylovulum psychrotolerans* S1L and *Methylomonas paludis* S2AM, from hypoxic water column layers of O_2_-stratified boreal lakes located in Southern Finland (Table 1, pictures on the colonies and cells in Fig. S1). The strains’ isolation, genome sequencing, and phylogenetic assignment were described previously (13). The genes in their genomes and representative MAGs of metagenomic operational taxonomic units representing *Methylomonas* spp. and *Methylovulum* spp. of boreal and subarctic lakes and ponds as well as one temperate lake and one tropical reservoir [MAGs assembled and taxonomically annotated by Buck et al. (14)] were predicted using Prodigal (v. 2.6.3) (15) and annotated according to Kyoto Encyclopedia of Genes and Genomes using KofamKOALA (https://www.genome.jp/tools/kofamkoala/; accessed 27 February 2023) (16). We specifically focused on the key genes encoding enzymes involved in organic acid and H_2_ production. The optimum growth conditions of the strains were determined in batch tests at different pH, temperatures, and nitrogen sources (see detailed methods in Supplementary Information) (Table 1; Fig. S2 and S3). In addition, the isolates’ capacity to generate organic acids was demonstrated in specific batch tests (six bottles per strain) as described in Khanongnuch et al. (10). Briefly, S1L and S2AM were grown in nitrate mineral salt medium and incubated at 23°C. For three bottles in the experimental setup, the initial headspace [containing 20% CH_4_ + 80% air (vol/vol)] was replenished with the original headspace content at days 10 and 14 for S1L and S2AM, respectively. As a control, the remaining experimental bottles were left without headspace replenishment, and the incubation was continued until days 20 and 34 for S1L and S2AM, respectively. During incubation, the cell growth, gaseous content, and organic acids were periodically monitored (see detailed methods in Supplementary Information) (Fig. 1).

**TABLE 1:** Characteristics of the gMOB isolates

Strain	*Methylovulum psychrotolerans* S1L	*Methylomonas paludis* S2AM	*Methylobacter* sp. S3L5C
Cell morphology	Cocci	Rods	Cocci
Cell size (µm)	1.0–1.8 diameter	0.7–1.2 × 1.4–3.0	1.7–4.0 diameter
Optimal temperature (growth) (°C)^*a*^	20–24 (4–30)	15–27 (0.2–30)	8–12(0.1–20)
Optimal pH (growth)	7.4 (4.7–8.3)	6.0–6.9 (5.0–7.5)	6.0–7.3 (6.0–8.3)
*nif* Gene	Yes	Yes	Yes
Motility^*b*^	–	–	–
Pigmentation	Pale pink	Pale pink	–
Excreted organic acid compounds	Acetate, formate, malate, and succinate	Acetate, formate, malate, succinate, and lactate	Acetate, formate, malate, and propionate
Carbon conversion efficiency of consumed methane into total accumulated organic acids^*c*^			
Acetate-C	0.7	2.7	2.4
Formate-C	0.1	0.4	<0.1
Malate-C	0.1	0.1	<0.1
Succinate-C	<0.1	0.1	–
Lactate-C	–	0.3	–
Propionate-C	–	–	0.1
	0.9%	3.6%	2.5%
Source	Lake water layer (Lovojärvi, Finland)	Lake water layer (Alinen Mustajärvi, Finland)	Lake water layer (Lovojärvi, Finland)
Reference	This study	This study	Khanongnuch et al. 2022 (10)

^*a*^ Based on the temperature test, S1L and S2AM are psychrotolerant, while S3L5C is psychrophilic.

^*b*^ –, not detected.

^*c*^ Organic acid accumulation at the end of the test with CH4 and air replenishment. See the calculation in supplemental data for Fig. 1 in the sections C,E-S1L-GC and D,F-S2AM-GC.

Strains S1L and S2AM were psychrotolerant and enabled to use of nitrate and ammonium as nitrogen sources (Table 1; Fig. S2 and S3). Both strains produced acetate, formate, malate, and succinate, while S2AM also produced lactate (up to 0.2 µM) in the subsequent specific tests to demonstrate their organic acid production (Table 1; Fig. 1G through J). Similar as noticed for S3L5C as reported by Khanongnuch et al. (10), acetate was the most prominent metabolite, up to 0.8 µM and 2.9 µM, for S1L and S2AM, respectively, in these specific batch tests. It was followed by formate, up to 0.1 µM and 0.9 µM, for S1L and S2AM, respectively, while the other products had lower concentrations, < 0.1 µM (Fig. 1G–J). The average consumed O_2_/CH_4_ ratio (~1.0) was below the stoichiometric ratio in aerobic CH_4_ oxidation (Fig. 1E and F). This indicates that O_2_-limited CH_4_ oxidation (during hypoxic conditions) initiated the accumulation of organic acids (4, 10). For S1L, the growth and accumulation of organic acids were generally higher in the treatment with headspace gases replenished (Fig. 1A, G and I; Fig. S4A) (growth: *P* < 0.01, organic acids: *P* < 0.01, see Supplementary data for Fig. 1 and Fig. S4), agreeing with results from our previous study of *Methylobacter* sp. S3L5C (10). For S2AM, the gas replenishment did not improve growth or organic acid accumulation (Fig. 1B, H and J; Fig. S4B) (growth: *P* = 0.57, organic acids: *P* = 0.51, see Supplementary data for Fig. 1; Fig. S4), likely due to the high viscosity visually observed in the liquid medium, causing mass transfer limitation on CH_4_ uptake.

The genes encoding putative enzymes driving the organic acid production were found in the genomes of both strains (Table S1; Fig. S5). As further proof of functions under fermentative conditions, both strains contained genes encoding H_2_-producing enzymes (Table S1), as did S3L5C (10). Surprisingly, lactate was observed during incubation of S2AM (Fig. 1H and J); however, its genome did not encode an identifiable lactate dehydrogenase. It is possible that lactate excretion is from methylglyoxal/2-oxopropanal detoxification generally occurring in microorganisms (17, 18). This detoxification to D-lactate was potentially carried out by the products of the *gloA* and *gloB* genes found in S2AM (Table S1), responding with the observation in other methanotrophs (19, 20). However, this observation requires further experimental validations, and the methylglyoxal formation in methanotrophs has not been elucidated (20). Our MAG analyses also indicate that the genetic potential of *Methylomonas* spp. and *Methylovulum* spp. for organic acid and H_2_ production is widely dispersed in boreal and subarctic lakes and ponds (Finland, Sweden, and Canada) and also found within the temperate lake (Switzerland) and tropical reservoir (Puerto Rico) in the MAG data set (Table S1), similar as noticed for *Methylobacter* spp. in environmental samples (10 – 12).

Altogether, our experiments with gMOB strains representing three genera (Table 1) and MAG analyses demonstrate that the ability to convert CH_4_ to various organic acids is a prevalent trait among lake and pond gMOB. Hence, gMOB are important mediators in incorporating CH_4_-carbon into microbial food webs of freshwater lake and pond ecosystems.

## Supplementary Material

Reviewer comments

## Data Availability

The research data are available in the supplemental data sets (see Supplemental Material).

## SUPPLEMENTAL MATERIAL

Supplemental data for Fig. 1 spectrum.01742-23-s0001.xlsx Supporting data related to Fig. 1.

Supplemental data for Fig. S2 spectrum.01742-23-s0002.xlsx Supporting data related to Fig. S2.

Supplemental data for Fig. S3 spectrum.01742-23-s0003.xlsx Supporting data related to Fig. S3.

Supplemental data for Fig. S4 spectrum.01742-23-s0004.xlsx Supporting data related to Fig. S4.

Supplemental information spectrum.01742-23-s0005.docx Supplemental methods and Fig. S1 to S5.

Table S1 spectrum.01742-23-s0006.xlsx Key genes encoding enzymes involved in organic acid and H_2_ production in *Methylomonas paludis* S2AM and *Methylovulum psychrotolerans* S1L, as well as representative MAGs of mOTU affiliated with *Methylomonas* and *Methylovulum*.

OPEN PEER REVIEW reviewer-comments.pdf An accounting of the reviewer comments and feedback.

ASM does not own the copyrights to Supplemental Material that may be linked to, or accessed through, an article. The authors have granted ASM a non-exclusive, world-wide license to publish the Supplemental Material files. Please contact the corresponding author directly for reuse.
