# Supplementary material for: Conversion of methane to organic acids is a widely found trait among gammaproteobacterial methanotrophs of freshwater lake and pond ecosystems
Source: Microbiol Spectr. 2023 Oct 20;11(6):e01742-23. doi: 10.1128/spectrum.01742-23 (PMC10715148; doi:10.1128/spectrum.01742-23)
Supplement: Supplemental information — Supplemental methods and Fig. S1 to S5. [file spectrum.01742-23-s0005.docx]

**Supplementary data for the manuscript includes:**

1. Supplementary Information (including Supplementary Material and methods and Figures S1-S5, see below in this document)
2. Supplementary_TableS1.xlsx - file (Table S1, submitted as a separate file from other Supplementary Information)
3. Supplementary data files including data used to generate the figures, i.e.,
   1. Supplementary data for Figure1.xlsx
   2. Supplementary data for FigureS2-S1L.xlsx
   3. Supplementary data for FigureS3-S2AM.xlsx
   4. Supplementary data for FigureS4.xlsx

**Supplementary information**

**Material and methods**

**1. Cultivation in different temperatures, pH, and nitrogen sources**

The growth experiments at different temperatures (0-34°C) and pH (4.7-8.3) were performed in 25-ml vials containing 5 ml of nitrate mineral salt (NMS; 10 mM nitrate) medium (1) inoculated with active cells at initial optical density (OD_600nm_) of 0.02. The headspace contained 20% CH_4_ and 80% air with atmospheric pressure. The growth in different nitrogen sources was performed in a 25 ml vial containing NMS (10 mM nitrate) and ammonium mineral salt (AMS) media (10 mM ammonium) (pH 6.8) and statically incubated at 20°C, except for the strain S2AM, for which the different dilutions of AMS media were tested, including 10, 5, 2, and 1 mM, due to no cell growth observed in the initial AMS medium (10 mM ammonium). The gaseous content in the headspace and liquid culture were periodically monitored.

**2. Analytical methods**

In all batch tests, the gas composition of headspace was measured using a gas chromatography-thermal conductivity detector column (GC-TCD) equipped with a Carboxen-1000 60/80 column (Agilent Technologies, USA) with similar conditions as described in Khanongnuch et al. (2022)(2). Briefly, helium was used as the carrier gas at 30 ml min^-1^, and the injector and detector temperatures were 155 °C. For measuring CH_4_, CO_2_, O_2,_ and N_2_, the oven temperature was held at 35°C for 3.75 min, then increased at 30°C min^-1^ until 150°C and held for 3 min. For measuring only CH_4_ and CO_2_, the oven temperature was held at 150°C for 3 min.

The liquid culture (1 ml) was collected for measuring OD_600nm_ and pH using an Ultrospec 500 pro spectrophotometer (Amersham Biosciences, UK) and a pH 330i portable meter (WTW, Germany), respectively, as described in Khanongnuch et al. (1). The culture was then centrifuged (15 min at 2700 × g) and the supernatant was filtered through a 0.2 μm membrane (Chromafil® Xtra PET 20/25, Macherey-Nagel, Germany) to determine the organic acid composition using a Shimadzu high-performance liquid chromatography equipped with Rezex RHM-Monosaccharide H^+^ (8%) column (Phenomenex, USA) and the conditions were adapted from Okonkwo (3). Specifically, the mobile phase consisted of 0.01 N H­_2_SO_4_ with a flow rate of 0.4 mL min^-1^ and column temperature was set at 70 °C. Standard solutions were prepared using sodium acetate, sodium formate, sodium succinate, sodium lactate, and sodium malate with concentrations ranging from 0 to 5 mM.

**References**

1. Khanongnuch R, Mangayil R, Svenning MM, Rissanen AJ. Characterization and genome analysis of a psychrophilic methanotroph representing a ubiquitous Methylobacter spp. cluster in boreal lake ecosystems. ISME COMMUN. 2022 Sep 19;2(1):85.

2. Khanongnuch R, Mangayil R, Santala V, Grethe Hestnes A, Marianne Svenning M, Rissanen AJ. Batch Experiments Demonstrating a Two-Stage Bacterial Process Coupling Methanotrophic and Heterotrophic Bacteria for 1-Alkene Production From Methane. Frontiers in Microbiology. 2022;13:874627.

3. Okonkwo O, Lakaniemi AM, Santala V, Karp M, Mangayil R. Quantitative real-time PCR monitoring dynamics of Thermotoga neapolitana in synthetic co-culture for biohydrogen production. International Journal of Hydrogen Energy. 2018 Feb 8;43(6):3133–41.

**Supplementary figures**

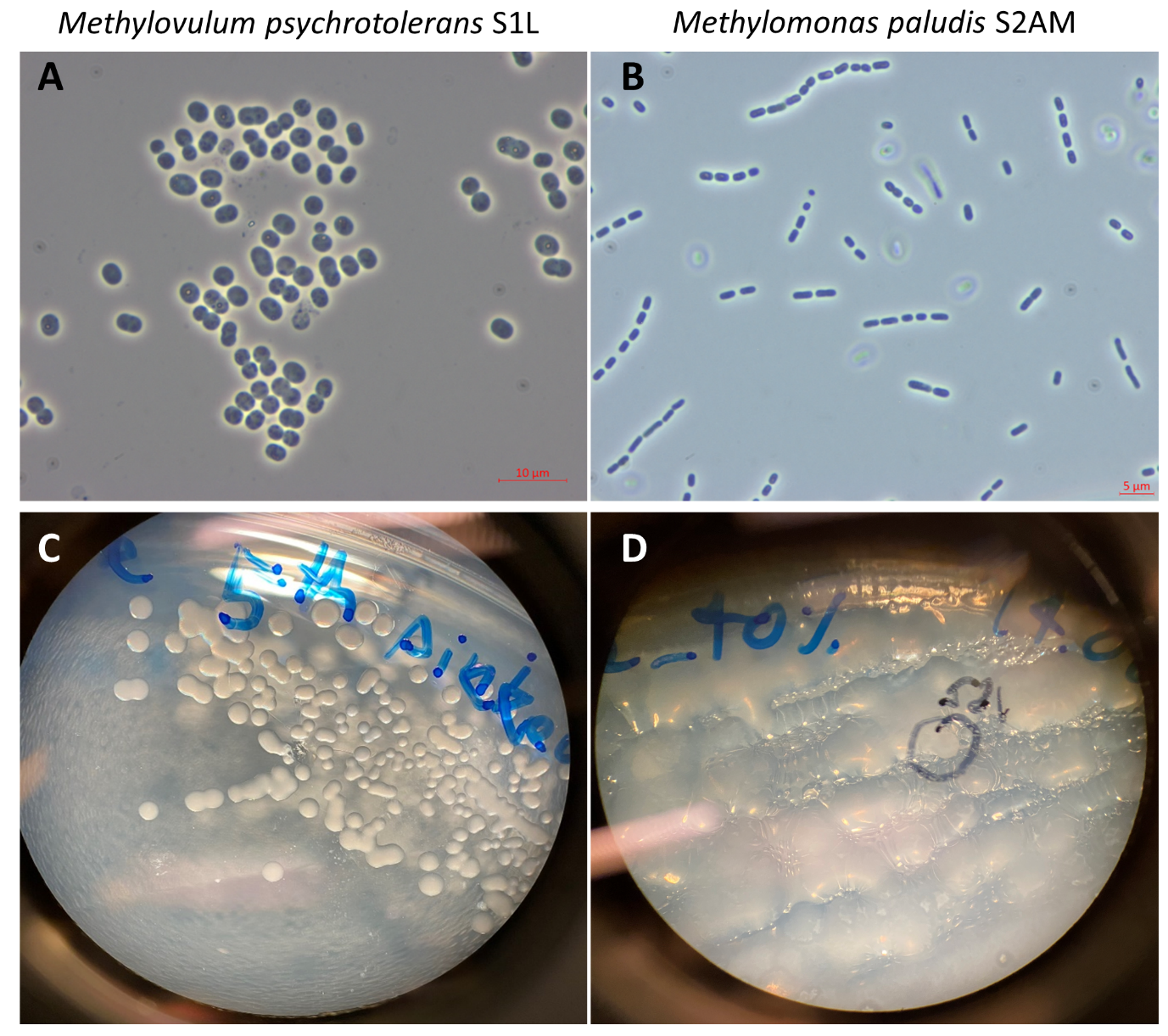


**Figure S1**. Cell and colony morphologies. (A, B) Light microscopy images and (C, D) photographs of colonies under a stereo microscope of *Methylovulum psychrotolerans* S1L and *Methylomonas paludis* S2AM, respectively.

**Fig. S2**

**
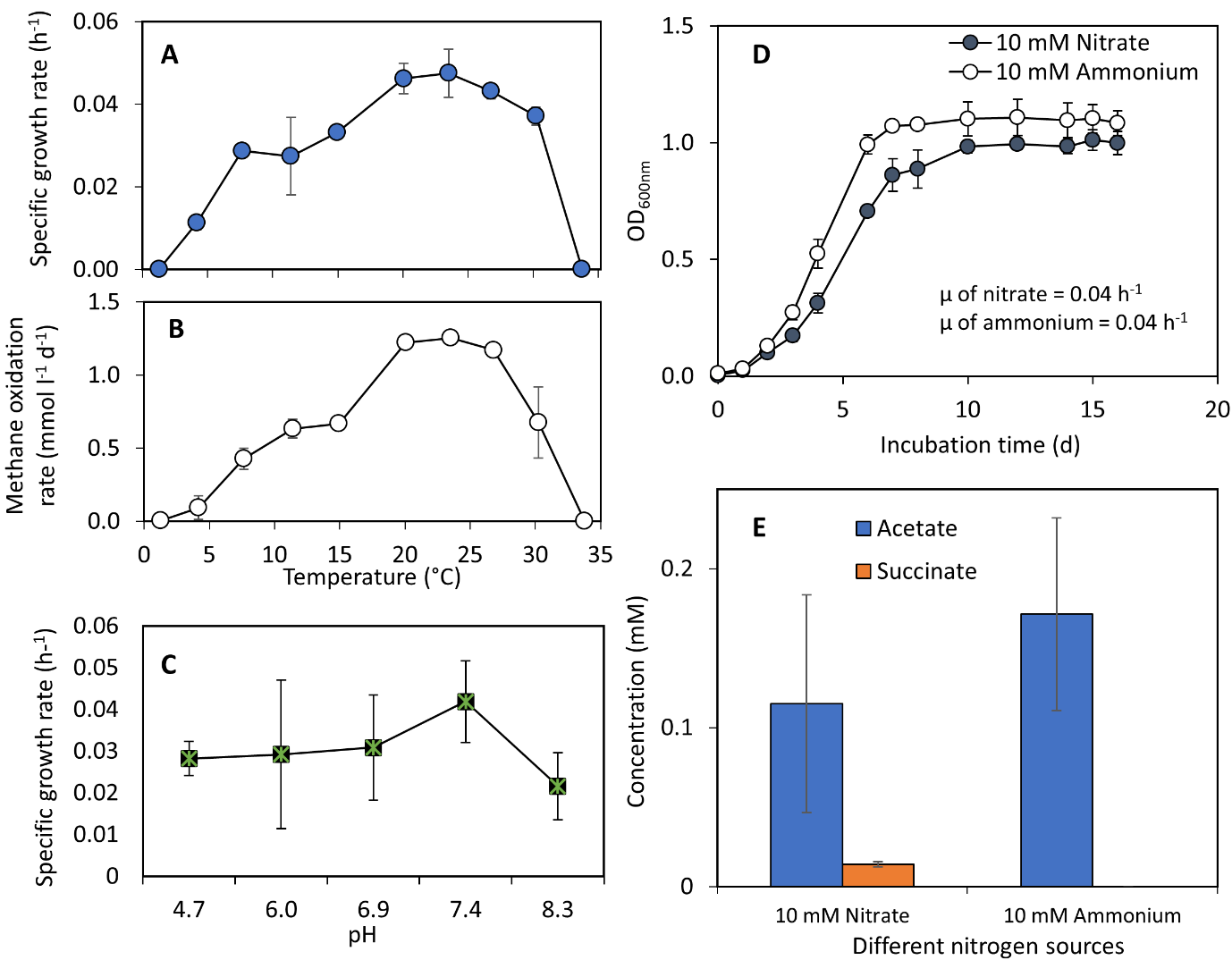
**

**Figure S2**. Characterization of *Methylovulum psychrotolerans* S1L with different temperature, pH, and nitrogen sources in batch cultivation. (A, B) Specific growth rate and methane oxidation rate at different temperatures. (C) Specific growth rate at different pH. (D, E) OD_600nm_ (and specific growth rate) and organic acid concentrations in different nitrogen sources (i.e., nitrate and ammonium). The error bars represent the standard deviation between biological duplicate samples in temperature gradient tests (A, B) and among the biological triplicate samples in tests for different pH and nitrogen sources (C-E).


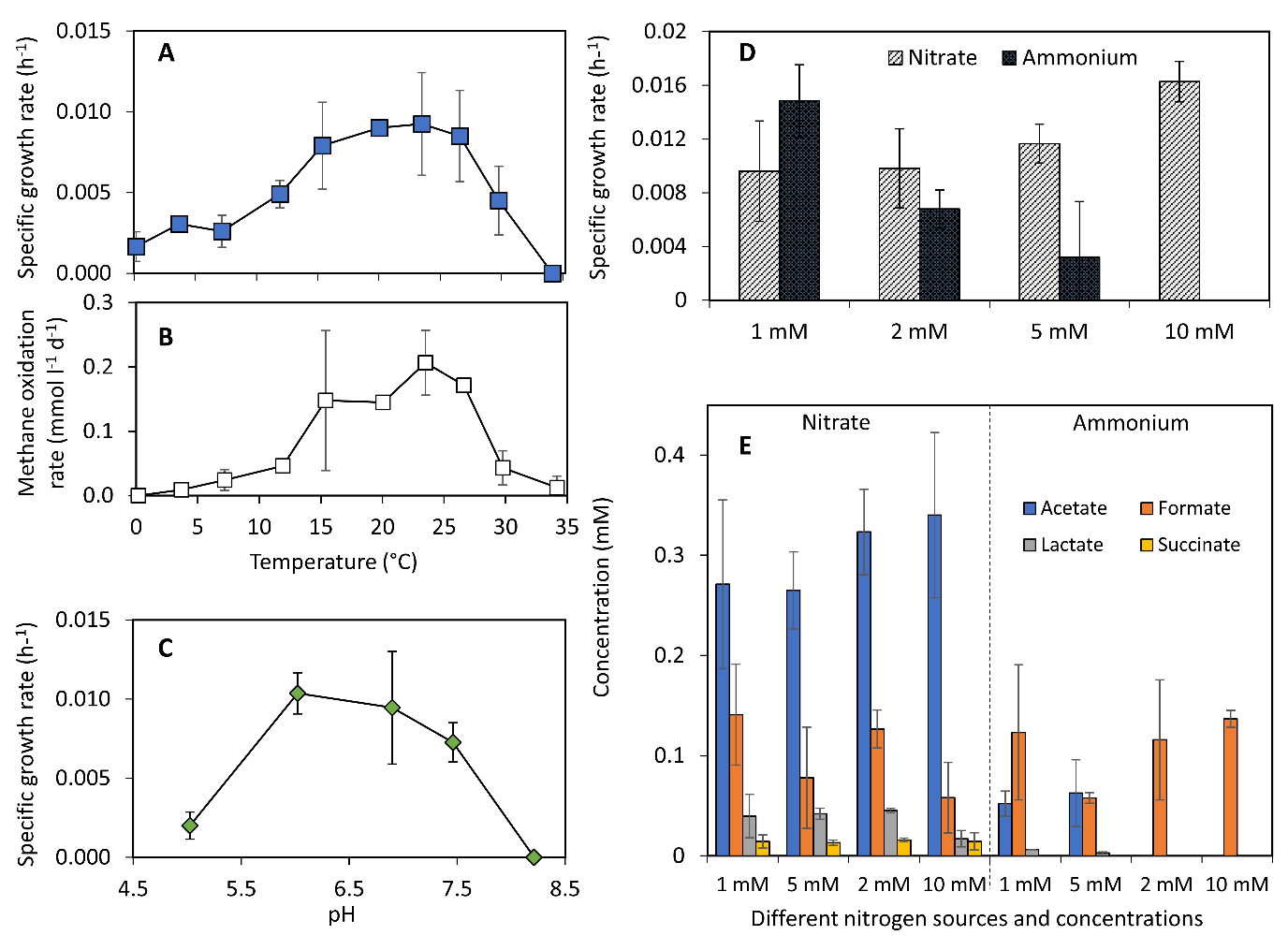


**Figure S3**. Characterization of *Methylomonas paludis* S2AM with different temperature, pH, and nitrogen sources in batch cultivation. (A, B) Specific growth rate and methane oxidation rate at different temperatures. (C) Specific growth rate at different pH. (D, E) specific growth rate and organic acid concentrations in different nitrogen sources (i.e., nitrate and ammonium) with different concentrations. The error bars represent the standard deviation between biological duplicate samples in temperature gradient tests (A, B) and among the biological triplicate samples in tests for different pH and nitrogen sources (C-E).


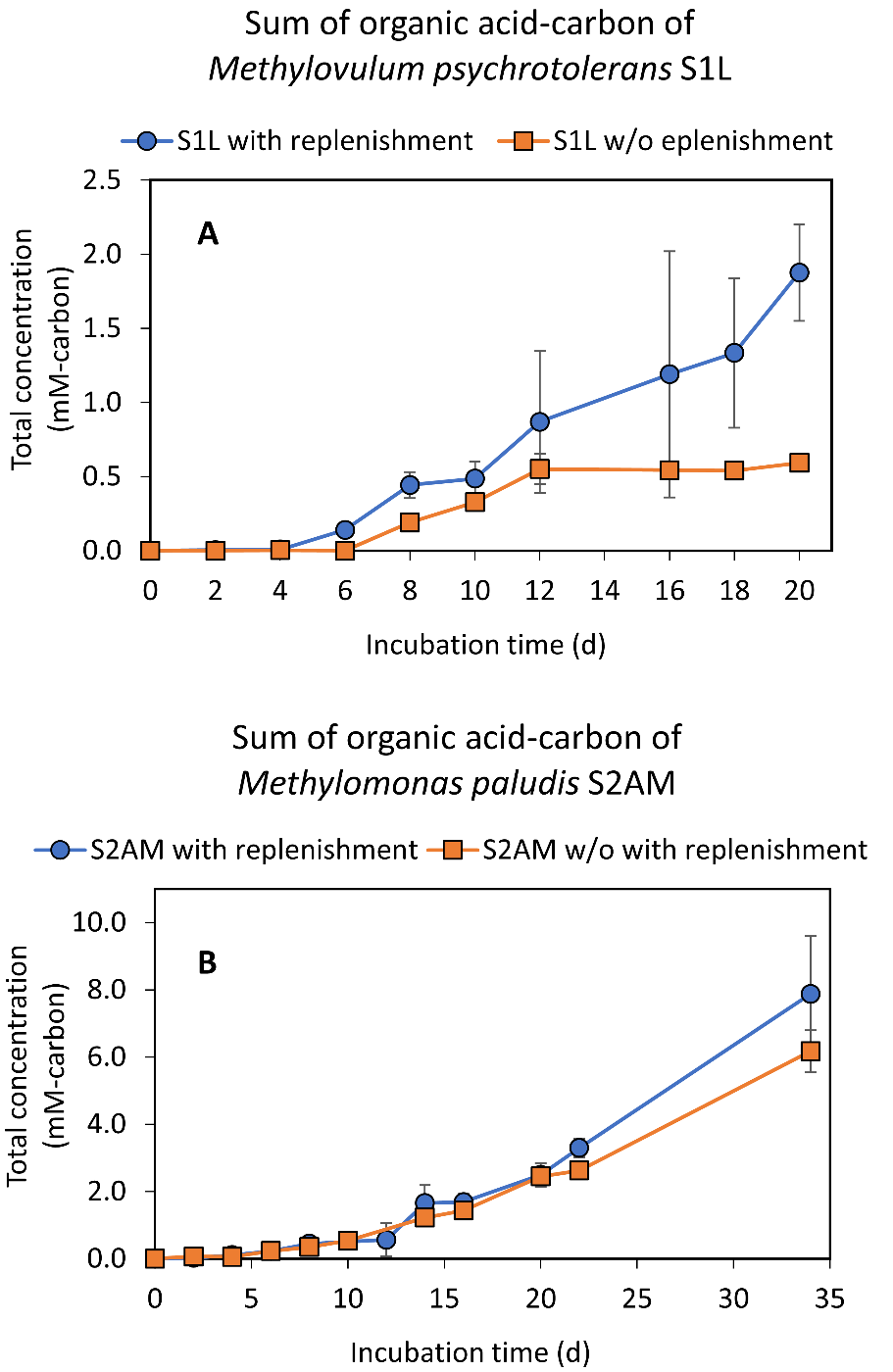


**Figure S4**. Total organic acid-carbon concentration in the liquid medium during 20- and 34-day incubation for A) *Methylovulum psychrotolerans* S1L and B) *Methylomonas paludis* S2AM, respectively, in both tests with and without CH_4_ and air replenishment. The error bars represent the standard deviation among the biological triplicate samples.


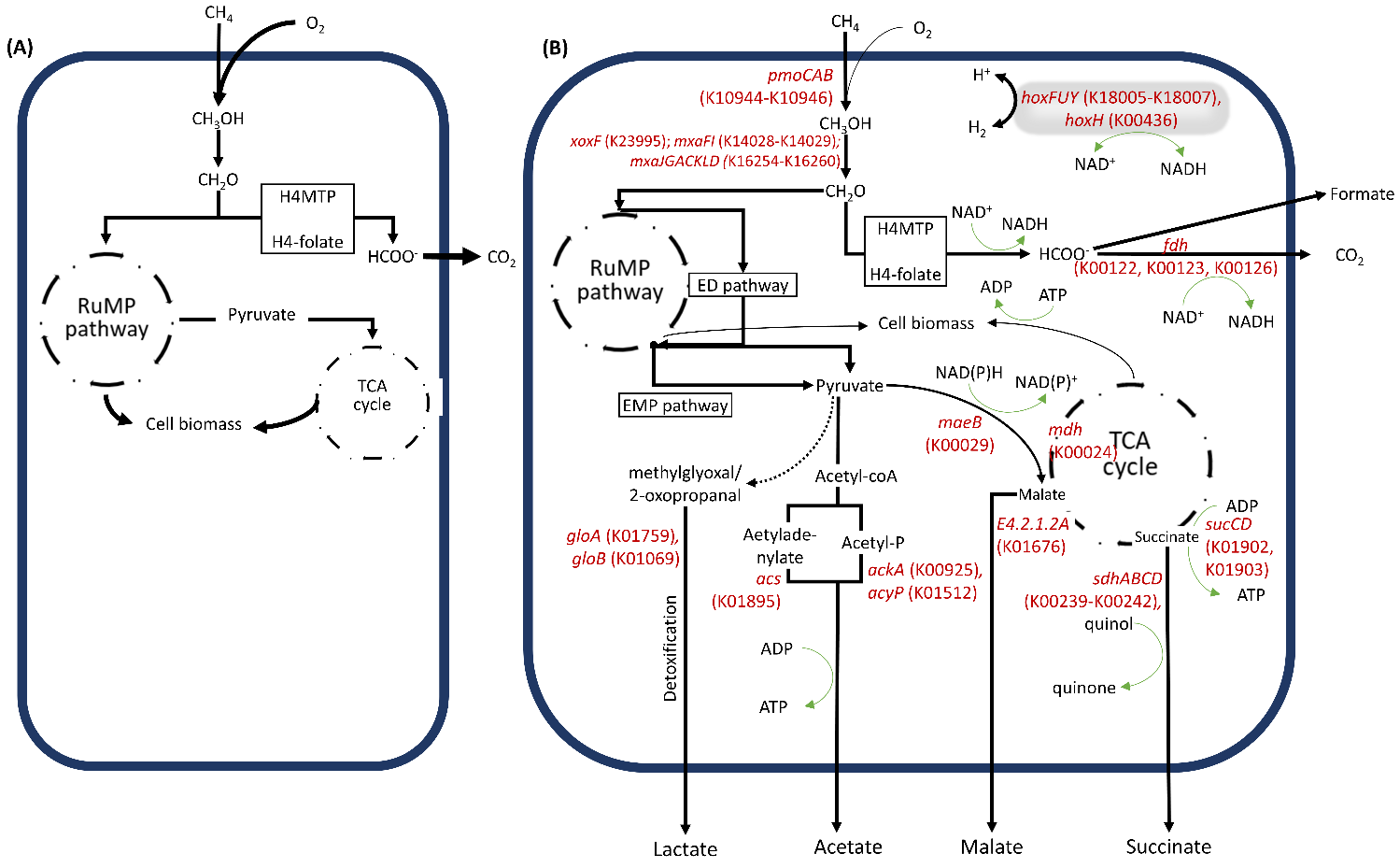


**Figure S5**. Proposed pathway for organic acid production during methane oxidation under A) oxic and B) hypoxic conditions in aerobic gammaproteobacterial methanotrophs (gMOB), i.e., *Methylovulum psychrotolerans* S1L and *Methylomonas paludis* S2AM. The genes annotated for involvement in organic acid production, as found in the genomes of both S1L and S2AM strains, are listed in Supplementary Table S1.
